# Impact of left atrial myopathy and post-ablation remodeling on quality of life: a DECAAF II sub-analysis

**DOI:** 10.1007/s10840-025-02002-1

**Published:** 2025-02-03

**Authors:** Ghassan Bidaoui, Han Feng, Nour Chouman, Ala Assaf, Chanho Lim, Hadi Younes, Mayana Bsoul, Christian Massad, Francisco Tirado Polo, Yishi Jia, Yingshou Liu, Abboud Hassan, William Rittmeyer, Mario Mekhael, Charbel Noujaim, Amitabh C. Pandey, Swati Rao, Omar Kreidieh, Nassir F. Marrouche, Eoin Donnellan

**Affiliations:** 1 Tulane Research Innovation for Arrhythmia Discovery, Tulane University School of Medicine, 1430 Tulane Avenue, New Orleans, LA 40130 USA; 2 Cleveland Clinic, Cleveland, OH USA; 3 Southeast Louisiana Veterans Health Care System, New Orleans, LA USA

**Keywords:** Atrial fibrillation, Atrial remodeling, Catheter ablation, Quality of life

## Abstract

**Background:**

Atrial fibrillation (AF) is associated with adverse remodeling of the left atrium (LA). The impact of the extent of atrial myopathy and post-ablation remodeling on quality-of-life (QoL) outcomes has not been studied.

**Objective:**

The aim of our study was to investigate the association between atrial myopathy and post-ablation remodeling on quality-of-life outcomes in patients with persistent AF.

**Methods:**

We conducted an analysis of DECAAF II participants who underwent late-gadolinium enhancement MRI (LGE-MRI) before and after AF ablation. We assessed atrial myopathy and post-ablation atrial remodeling, scar formation, and fibrosis coverage with ablation. QoL metrics were assessed using the Short Form Survey (SF-36) and Atrial Fibrillation Severity Scale (AFSS). Uni- and multivariable regression models were developed for this analysis.

**Results:**

Six hundred thirteen patients with persistent AF were included in our analyses. At baseline, AFSS burden and total AFSS score were 18.94 ± 7.35 and 12.24 ± 8.17, respectively. Following ablation, all QoL and AFSS metrics improved in both the pulmonary vein isolation (PVI) and MRI-guided fibrosis ablation groups. On average, one unit of post-ablation reduction in left atrial volume index (LAVI) was associated with an improvement of 0.085 in total AFSS score (*p* = 0.001), 0.01 in shortness of breath with activity (*p* < 0.001), 0.15 in AF burden (*p* < 0.001), − 0.016 in global well-being (*p* = 0.018), 0.519 in health change (*p* < 0.001), 0.19 in vitality (vitality (*p* = 0.01), and 0.27 in physical functioning (*p* = 0.001). Baseline fibrosis and residual fibrosis post-ablation were associated with improved vitality and general health.

**Conclusion:**

Atrial myopathy and post-ablation atrial remodeling significantly impact QoL in patients with persistent AF undergoing ablation.

**Graphical abstract:**

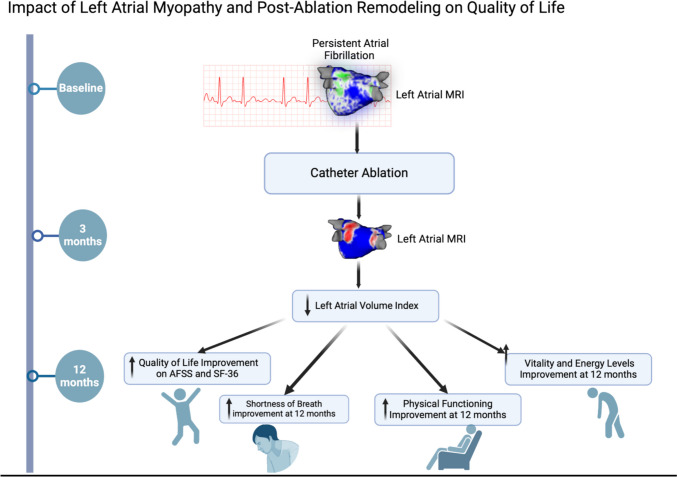

**Supplementary Information:**

The online version contains supplementary material available at 10.1007/s10840-025-02002-1.

## Introduction

Atrial fibrillation (AF) is the most common arrhythmia in developed countries [1]. Atrial myopathy describes adverse structural, electrical, and functional remodeling of the atrium [2]. A bidirectional relationship exists between AF and atrial myopathy. For instance, atrial interstitial fibrosis results in conduction heterogeneity, providing a substrate for reentry and increasing the inducibility, propagation, and perpetuation of AF [3, 4]. Sequelae of rapid atrial depolarization resulting from AF include myocyte apoptosis and accelerated fibrosis due to increased intracellular calcium accumulation [5].

In addition to increasing rates of cerebrovascular accident (CVA), congestive heart failure (CHF), and mortality, AF has profound quality-of-life (QoL) implications [6]. Catheter ablation (CA) of AF is associated with more improvements in QoL compared to other treatment modalities [7, 8]. Studies have demonstrated an inconsistent relationship between maintenance of sinus rhythm following ablation and QoL improvements [9]. Prior studies have demonstrated that CA is associated with a reduction in atrial volume independent of recurrence status [10].

Late-gadolinium enhancement cardiac MRI (LGE-MRI) represents the gold standard for noninvasively assessing atrial fibrosis, atrial volume, and CA-induced scar formation. These indices have been shown to be associated with post-ablation arrhythmia recurrence [11, 12]. To date, no studies have investigated the association between positive atrial remodeling following CA and QoL in patients with persistent AF. The aims of our study were threefold: (1) to investigate the association between baseline atrial myopathy and post-ablation QoL; (2) to study the structural atrial changes that occur following CA; and (3) to examine the association between post-ablation atrial remodeling and patient-reported QoL metrics.

## Methods

### Study design

The current study is a sub-analysis of the DECAAF II trial. A full discussion of the DECAAF II methods has been published previously in the literature [13, 14]. The DECAAF II trial was a prospective, randomized, investigator-initiated, multicenter, single-blinded trial randomizing patients with persistent AF to undergo either PVI or PVI plus MRI-guided ablation. Of the 843 randomized patients, 422 patients were in the PVI arm and 421 were in the PVI plus MRI-guided ablation arm. Patients were followed for 12–18 months using daily single-lead smartphone electrocardiography (ECG) recordings (ECG Check Device, Cardiac Designs Inc.) to assess for recurrence. Ambulatory monitoring and 12-lead ECG data performed as part of clinical care were also included. Written informed consent was obtained from all participants before study inclusion. The trial was approved by the ethics review committee at each participating center. This study was approved by the Tulane University Biomedical institutional review board.

### Patient population

Patients had to be at least 18 years old and have documented persistent AF without prior ablation. Persistent AF was defined as AF lasting 7 days or more on chart review or ECG. Exclusion criteria included patients with prior ablation or valvular cardiac surgery, contraindication for CMR or the use of beta-blockers, inability to be positioned appropriately in the MRI scanner, pregnant women, terminally ill patients, or those without smartphone capability. The current study included patients who underwent pre- and post-ablation LGE-MRI and completed QoL assessments prior to ablation and at least 12 months following ablation.

### Imaging protocol

All participants underwent LGE-MRI within 30 days prior to CA and again between 90 and 180 days following CA. Images were obtained 15 min following gadolinium administration using a high-resolution, three-dimensional, inversion-recovery-prepared, ECG-gated, respiratory-navigated, gradient-echo pulse sequence for LGE-MRI. The timing of image capture was carefully chosen to occur before atrial systole, during a period known to have minimal LA wall movement.

The Merisight delayed CMR protocol by MARREK, Inc. was used. The segmentation of the LA wall was done manually, and to identify atrial fibrosis, an intensity threshold was applied, ranging from two to three standard deviations above the average intensity of the normal tissues, as determined by experts. Baseline fibrosis measurement involved calculating the fibrosis amount in the LA wall before ablation and dividing it by the atrial surface area. The post-ablation scar was quantified by the percentage of the left atrium showing enhancement at a heightened LGE threshold, concealing pre-ablation fibrosis, thus representing the iatrogenic scar from CA. The CMR images from before and after ablation were aligned using affine registration for detailed examination, followed by deformable registration. A 3D map of LA fibrosis was then constructed with the Corview volume rendering tool. Residual fibrosis refers to the remaining fibrotic tissue in the atrium after subtracting areas overlaid by scarring, quantified as a percentage of the wall’s total volume [15].

### QoL assessment

QoL outcomes were measured in DECAAF II as a main efficacy outcome through the Toronto Atrial Fibrillation Symptom Severity (AFSS) (AFSS© Unity Health Toronto, 2001) [16] scale. The patients were required to submit an AFSS questionnaire at baseline (pre-ablation), at 3 months, and at 12 months post-ablation. The questionnaire at 12 months post-ablation serves as the primary QoL outcome assessment. The AFSS is a validated questionnaire that was developed to capture the severity of AF-associated symptoms. The questionnaire consists of 19 questions, and it assesses both objective and subjective dimensions of AF. It assesses AF symptoms both at rest and with activity such as palpitations, shortness of breath, dizziness, fatigue, and energy levels. For each symptom, the severity is rated from 0 to 5. The AFSS scores can range from 0 to 35, with 35 indicating the highest symptom severity. Moreover, the questionnaire assesses disease burden such as frequency, severity, and duration of episodes. The scores were calculated based on the “Scaling and Scoring version 1.0: August 2020” method shared by Mapi Research Trust Analysis and quantification of improvement. A cumulative measure of AF burden was obtained by combining the equally contributing frequency, duration, and severity of AF episodes. Each measure of these is given a score from 1 to 10,and the total score ranges from 3 to 30. Improvement for each individual component was defined as the difference between the post-ablation and baseline score.

The 36-item short-form health survey (RAND 36-Item Health Survey Version 1.0) questionnaire was another validated questionnaire that was used and administered at the same intervals in DECAAF II to assess multiple physical and mental health dimensions [17]. This form tackles eight health dimensions: pain, energy/fatigue, general health perception, health change, social functions, mental health, role limitations due to emotional problems, physical functioning, role limitations due to physical health, and general mental health. Scoring was performed using the validated RAND-36 scoring method shared by the International Resource Center for Health Care Assessment. Each item is scored on a scale from 0 to 100, with 100 representing the highest level of functioning possible. The improvement in these measures was calculated as the difference between the post-ablation and the baseline scores.

### Statistical analysis

Changes in QoL and symptom severity were assessed at 12 months compared with baseline using the SF-36 and AFSS questionnaires. The individual components and the total AFSS score were assessed separately for association with imaging indices such as baseline fibrosis, left atrial volume, left atrial volume index change, and residual fibrosis, among others. The beta coefficient (β) quantifies the expected change in the dependent variable (e.g., QoL metrics) for each one-unit increase in the independent variable. For instance, a β of + 5.0 suggests that a single-unit increase in the independent variable is associated with a 5-point rise in the dependent variable. The β’s sign indicates the direction of this relationship: a positive sign ( +) denotes a direct correlation, where the dependent and independent variables move in tandem, whereas a negative sign ( −) indicates an inverse relationship, meaning they move in opposite directions. Univariate regression analysis was performed to screen for any signal of significance between imaging parameters and QoL outcomes. Factors that were significantly associated with QoL change on univariable analysis were subsequently assessed in multiple linear regression models to control for clinically relevant confounders such as age, gender, arrhythmia recurrence, left atrial volume, baseline left ventricular ejection fraction (LVEF) and change in LVEF, baseline fibrosis, comorbidities, and treatment arm. Descriptive statistics for continuous and categorical variables were summarized as mean ± standard deviation and percentages, respectively. All statistical analyses were performed using R 4.2.0 (The R Foundation) [18] or SPSS 29 with a two-sided significance level of 0.05 by default [19].

## Results

### Baseline characteristics

**Fig. 1:**
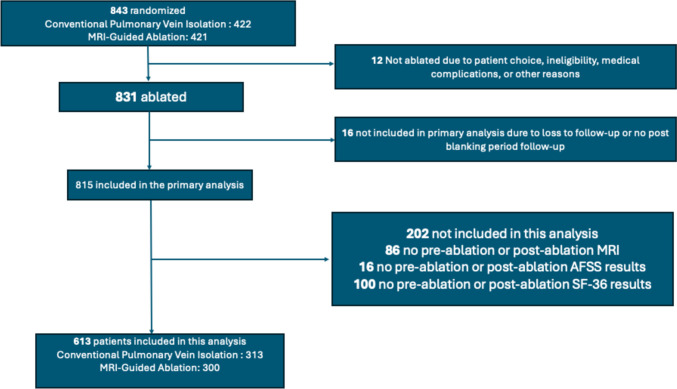
Patient inclusion and exclusion from analysis. Legend: Among 843 patients randomized to pulmonary vein isolation or MRI-guided ablation, 613 patients met the final inclusion criteria

A total of 613 patients with persistent AF were included in our analyses based on the exclusion criteria (Fig. 1). Of these, 313 had been randomized to the PVI arm and 300 had been randomized to the MRI-guided ablation arm. The baseline characteristics of these patients including their medication use, comorbidities, and baseline imaging indices are shown in Tables 1 and 2. The mean age of the study cohort was 62.2 ± 9 years and females constituted 22.7% of the study population. A total of 60% of the study cohort had hypertension and 17.8% had CHF. Eighty-nine percent of the patients had a history of cardioversion and around 56% were taking antiarrhythmic medications.

**Table 1:** Baseline characteristics of the study cohort

Variables Mean (SD)/counts (%)	Conventional PVI (*N* = 313)	PVI + fibrosis-guided ablation (*N* = 300)	Total (*N* = 613)	*p*-value
Age (years)	62.54 (9.22)	61.86 (8.80)	62.21 (9.02)	0.20
Sex (female)	72 (23.0%)	67 (22.3%)	139 (22.7%)	0.84
BMI (kg/m^2^)	31.85 (6.79)	31.44 (6.03)	31.65 (6.43)	0.46
Time from diagnosis to ablation (months)	36.25 (47.03)	35.87 (43.24)	36.06 (45.18)	0.67
Congestive heart failure (%)	46 (14.7%)	63 (21.0%)	109 (17.8%)	0.041
Hypertension (%)	188 (60.1%)	180 (60.0%)	368 (60.0%)	0.99
Diabetes mellitus (%)	34 (10.9%)	29 (9.7%)	63 (10.3%)	0.63
History of stroke (%)	28 (8.9%)	21 (7.0%)	49 (8%)	0.37
Smoking (%)	122 (39.0%)	112 (37.3%)	234 (38.2%)	0.68
Coronary artery disease (%)	37 (11.8%)	40 (13.3%)	77 (12.6%)	0.57
Mitral valve disease (%)	20 (6.4%)	17 (5.7%)	37 (6%)	0.71
Hyperlipidemia (%)	104 (33.2%)	113 (37.7%)	217 (35.4%)	0.25
Antiarrhythmic medications (%)	137 (43.8%)	146 (48.7%)	283 (46.2%)	0.22
Beta-blockers (%)	234 (74.8%)	228 (76.0%)	462 (75.4%)	0.72
Calcium channel blockers (%)	72 (23.0%)	68 (22.7%)	140 (22.8%)	0.92
ACE inhibitors (%)	79 (25.2%)	90 (30.0%)	169 (27.6%)	0.19
ARB inhibitors (%)	82 (26.2%)	77 (25.7%)	159 (25.9%)	0.88
Aldosterone inhibitors (%)	26 (8.3%)	29 (9.7%)	55 (9%)	0.56
Statins (%)	93 (29.7%)	109 (36.3%)	202 (33%)	0.081
Anticoagulation (%)	301 (96.2%)	290 (96.7%)	591 (96.4%)	0.74
History of cardioversion (%)	262 (83.7%)	253 (84.3%)	515 (84%)	0.83
Failed antiarrhythmic treatment (%)	175 (55.9%)	169 (56.3%)	344 (56.1%)	0.92

*BMI*, body mass index; *ARB*, angiotensin receptor blocker

**Table 2:** MRI indices and post-ablation change

Variables	Total (*N* = 613)	Conventional PVI (*N* = 313)	PVI + fibrosis-guided ablation (*N* = 300)	*p*-value
Baseline LAVI (mm^3^/m^2^)	61.79 (± 18.58)	61.64 (± 18.06)	61.96 (± 19.13)	0.990
Post-ablation LAVI (mm^3^/m^2^)	51.08 (± 15.91)	52.22 (± 16.13)	49.89 (± 15.59)	0.068
LAVI change	10.70 (± 12.28) mm^3^/m^2^	9.38 (± 11.85) mm^3^/m^2^	12.06 (± 12.58) mm^3^/m^2^	0.0084
Baseline fibrosis (%)	18.43 (± 7.19)	18.78 (± 7.37)	18.06 (± 6.99)	0.29
Covered fibrosis	0.21 (± 0.11)	0.19 (± 0.11)	0.24 (± 0.11)	< 0.001
Scar formed (%)	9.58 (± 5.05)	8.37 (± 4.19)	10.85 (± 5.54)	< 0.001
Residual fibrosis (%)	14.59 (± 6.31)	15.35 (± 6.63)	13.79 (± 5.87)	0.006

*LAVI*. left atrial volume index; *PVI*, pulmonary vein isolation; *MRI*, magnetic resonance imaging

### CMR indices

Atrial myopathy, atrial remodeling, and ablation-induced scar formation indices are shown in Table 2. At baseline, LAVI was 61.79 ± 18.58 mm^3^/m^2^. LAVI change from pre- to post-ablation in the whole cohort was 10.7 ± 12.8 mm^3^/m^2^ and was significantly higher in the MRI-guided ablation arm (9.38 vs. 12.06 mm^3^/m^2^, *p* < 0.001).

We also assessed baseline fibrosis, ablation-induced scar, and fibrosis coverage with ablation lesions. Baseline fibrosis was not significantly different in the treatment arms with an average baseline fibrosis of 18.43 ± 7.19%. Ablation-induced scar on post-ablation MRI constituted around 9.58 ± 5.05% of the left atrium. On average, 25% of fibrosis was covered in the MRI-guided arm compared to 19% in the PVI arm (*p* < 0.001).

### Recurrence

Nearly half of the total cohort experienced the primary outcome of recurrence (*n* = 334, 54.5%). Consistent with the findings of the main trial, there was no significant difference in recurrence rates between patients who underwent conventional pulmonary vein isolation (PVI) ablation and those who underwent additional fibrosis-guided ablation (168, 46.3% vs. 134, 44.7%; *p* = 0.680). The average time to recurrence after the blanking period was 290 ± 191.94 days, with no significant difference observed between the two treatment arms (291.13 ± 189 days vs. 288.9 ± 194 days).

### QoL improvement

AFSS burden and total AFSS score were 18.94 ± 7.35 and 12.24 ± 8.17 at baseline, respectively (Table 3). All AFSS and SF-36 QoL items improved following CA compared to pre-ablation assessment **(**Tables 3 and 4**)** both in the PVI and fibrosis-guided ablation arms. As shown in Table 4, for all subscales of the AFSS and SF-36, there was no significant difference in improvement between both treatment arms. On univariable analysis, multiple associations between LGE-MRI indices and QoL outcomes were observed **(**Supplementary Table 1**).** A lower pre-ablation LAVI was associated with post-ablation improvements in AF frequency (*β* = 0.03, *p* < 0.001) and shortness of breath on activity (*β* = 0.009, *p* = 0.01).

**Table 3:** AFSS at baseline and at 12 months and post-ablation change

Variable	Baseline (*N* = 613)	12 months post-ablation (*N* = 613)	Post-ablation change in the total population (*N* = 613)	*p*-value	Post-ablation change in the PVI arm (*N* = 313)	Post-ablation change in the PVI + fibrosis-guided ablation arm (*N* = 300)	*p*-value (PVI vs. PVI + fibrosis-guided ablation arm)
AF global well-being	6.64 (± 2.04)	7.88 (± 1.62)	− 1.25 (± 2.09)	< 0.001	− 1.30 (± 2.09)	− 1.21 (± 2.08)	0.8363
AF frequency	7.20 (± 3.45)	2.25 (± 2.44)	4.96 (± 3.98)	< 0.001	4.75 (± 3.98)	5.19 (± 3.98)	0.2518
AF duration	8.16 (± 2.94)	5.03 (± 3.47)	3.10 (± 3.98)	< 0.001	2.99 (± 3.91)	3.22 (± 4.07)	0.5628
AF severity	5.00 (± 2.77)	4.45 (± 2.90)	0.55 (± 2.95)	< 0.001	0.55 (± 3.04)	0.55 (± 2.86)	0.7100
AF burden	18.94 (± 7.35)	8.53 (± 6.30)	10.42 (± 8.28)	< 0.001	10.19 (± 8.28)	10.65 (± 8.29)	0.4061
Palpitations	1.96 (± 1.69)	0.83 (± 1.24)	1.14 (± 1.84)	< 0.001	1.13 (± 1.82)	1.14 (± 1.87)	0.8205
Shortness of breath at rest	1.41 (± 1.54)	0.59 (± 1.06)	0.81 (± 1.55)	< 0.001	0.81 (± 1.57)	0.82 (± 1.52)	0.7601
Shortness of breath at activity	2.69 (± 1.66)	1.40 (± 1.49)	1.29 (± 1.73)	< 0.001	1.21 (± 1.72)	1.37 (± 1.74)	0.2969
Exercise intolerance	2.41 (± 1.71)	1.13 (± 1.40)	1.28 (± 1.77)	< 0.001	1.20 (± 1.80)	1.36 (± 1.74)	0.3296
Fatigue at rest	1.61 (± 1.50)	0.66 (± 1.09)	0.95 (± 1.56)	< 0.001	0.93 (± 1.58)	0.97 (± 1.55)	0.6032
Dizziness	1.31 (± 1.41)	0.60 (± 1.06)	0.72 (± 1.50)	< 0.001	0.74 (± 1.46)	0.69 (± 1.55)	0.5613
Chest pain	0.96 (± 1.30)	0.45 (± 0.95)	0.52 (± 1.32)	< 0.001	0.50 (± 1.40)	0.54 (± 1.23)	0.5907
Total AFSS score	12.24 (± 8.17)	5.62 (± 6.31)	6.62 (± 8.04)	< 0.001	6.49 (± 8.26)	6.76 (± 7.82)	0.5313

*AF*, atrial fibrillation; *AFSS*, Atrial Fibrillation Severity Scale

**Table 4:** SF-36 at baseline and at 12 months following ablation and post-ablation change

Variables	Baseline (*N* = 613)	12 months post-ablation (*N* = 613)	Post-ablation change in the total population (*N* = 613)	*p*-value (baseline vs. 12 months)	Post-ablation change in the PVI arm (*N* = 313)	Post-ablation change in the PVI + fibrosis-guided ablation arm (*N* = 300)	*p*-value (PVI vs. PVI + fibrosis-guided ablation arm)
Physical functioning	67.42 (± 25.36)	81.07 (± 22.93)	13.64 (± 25.43)	< 0.001	14.86 (± 25.61)	12.37 (± 25.22)	0.2569
Limitations due to physical health	52.35 (± 43.96)	77.05 (± 36.63)	24.70 (± 46.53)	< 0.001	23.83 (± 45.52)	25.61 (± 47.63)	0.8046
Limitations due to emotional problems	75.67 (± 37.28)	85.54 (± 31.94)	9.87 (± 39.93)	< 0.001	10.65 (± 39.34)	9.06 (± 40.58)	0.5466
Energy and fatigue	50.89 (± 22.86)	65.21 (± 20.40)	14.32 (± 23.37)	< 0.001	14.30 (± 23.36)	14.34 (± 23.41)	0.9122
Emotional well-being	72.69 (± 17.62)	78.50 (± 16.81)	5.82 (± 17.63)	< 0.001	6.11 (± 16.71)	5.51 (± 18.56)	0.8527
Social functioning	79.63 (± 22.65)	88.46 (± 19.10)	8.83 (± 24.06)	< 0.001	8.71 (± 23.30)	8.96 (± 24.86)	0.3763
Pain	78.95 (± 23.78)	82.91 (± 23.19)	3.96 (± 26.21)	< 0.001	3.75 (± 27.61)	4.19 (± 24.72)	0.9340
General health	59.42 (± 19.20)	69.95 (± 19.76)	10.53 (± 21.92)	< 0.001	10.18 (± 22.90)	10.88 (± 20.87)	0.2517
Health change	41.03 (± 24.98)	80.06 (± 23.63)	39.03 (± 33.59)	< 0.001	38.66 (± 34.25)	39.42 (± 32.94)	0.9798

Post-ablation improvement in LAVI was significantly associated with improvements in physical functioning (*β* =  − 0.1359, *p* = 0.035), vitality (*β* =  − 0.172, *p* = 0.035), general health (*β* =  − 0.114, *p* = 0.04), health change (*β* =  − 0.18, *p* = 0.034), AF burden (*β* =  − 0.056, *p* = 0.006), shortness of breath at rest (*β* =  − 0.009, *p* = 0.01), and dizziness (*β* =  − 0.008, *p* = 0.035) **(**Supplementary Table 2**).**

**Fig. 2:**
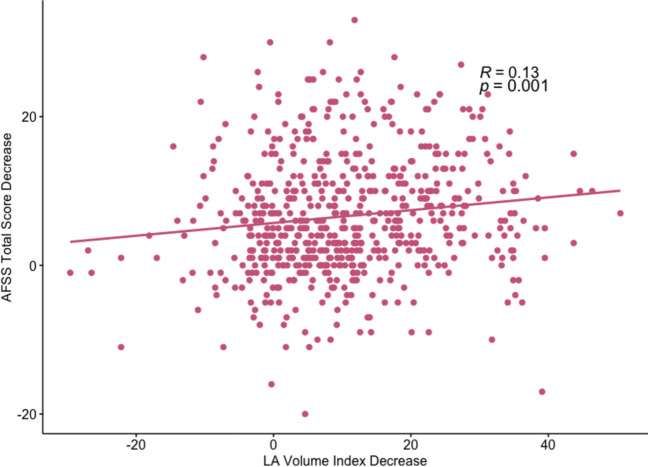
Correlation between LAVI change and AFSS change. Legend: This scatter plot displays the relationship between the decrease in left atrial (LA) volume index post-ablation and the reduction in AFSS (Atrial Fibrillation Severity Scale) score. The results suggest an improvement in the severity of atrial fibrillation symptoms with favorable remodeling

Post-ablation reduction in LAVI was significantly associated with total AFSS improvement (*β* = 0.085, *p* = 0.001) to a greater extent than baseline or post-ablation LAVI or substrate-related parameters such as baseline fibrosis or scar formation (Fig. 2).

**Fig. 3:**
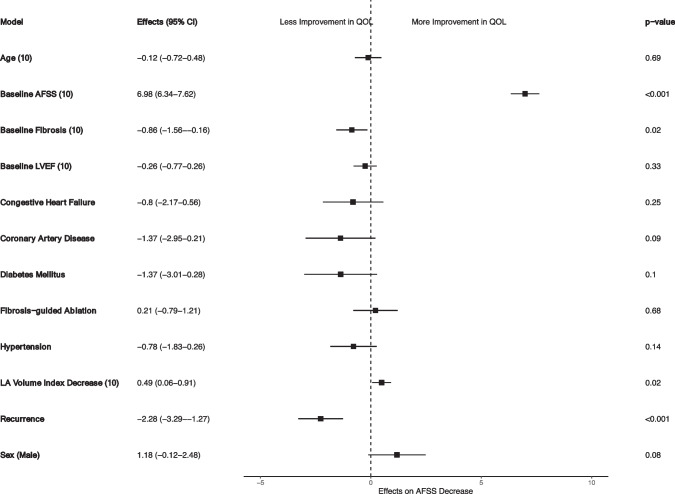
Multivariable linear regression model for AFSS change. Legend: This forest plot shows the results of the multivariable analysis for the outcome of AFSS score change. The multivariable analysis shows that baseline AFSS and LA volume index decrease are associated with improved AFSS score. Moreover, the association of LAVI decrease with AFSS improvement is still significant after controlling for the rest of the factors including the occurrence of atrial fibrillation recurrence post-ablation

In the multivariable linear regression model, various clinical confounders were included, such as patient demographics, comorbidities, treatment arm, and myopathy indices. The association between a reduction in LAVI and an improvement in AFSS remained significant, independent of potential confounding factors **(**Fig. 3**)**. Additionally, baseline fibrosis was significantly associated with attenuated AFSS improvement in this model. Baseline AFSS score emerged as the strongest predictor of improvement, indicating that patients with more severe symptoms at baseline experienced the greatest benefit.

Furthermore, atrial fibrillation recurrence was significantly associated with less symptom improvement but did not negate the significant effects of LAVI reduction and baseline fibrosis. LAVI reduction was also linked to improvements in specific metrics of the AFSS questionnaire, including global well-being (*β* =  − 0.0164, *p* = 0.018), AF frequency (*β* = 0.108, *p* < 0.001), and AF burden (*β* = 0.15, *p* < 0.001).

With respect to improvement in outcomes measured by the SF-36 questionnaire, LAVI decrease was associated with improvements in physical functioning (*β* = 0.27, *p* = 0.001), limitations due to physical health (*β* = 0.53, *p* < 0.001), vitality (*β* = 0.197, *p* = 0.01), and patient perspective on health change (*β* = 0.519, *p* < 0.001).

**Fig. 4:**
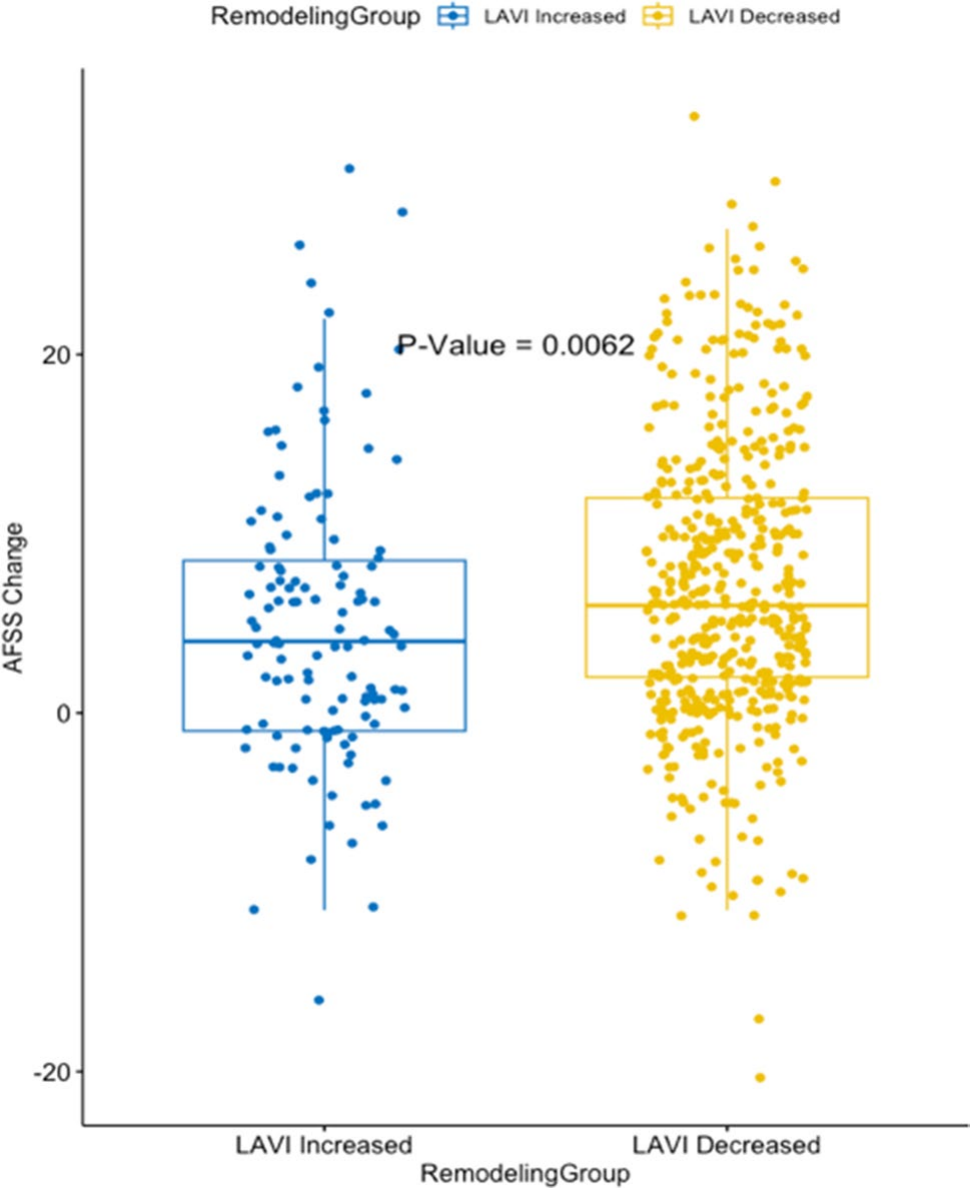
Change in AFSS based on increase or decrease in LAVI. Legend: This box plot compares the improvement in AFSS scores between two patient groups: those with an increased left atrial volume index (LAVI) and those with a decreased LAVI after ablation. More symptom severity improvement is seen in patients who had a decrease in LAVI (favorable remodeling) compared to patients with an increase in LAVI post-ablation

Baseline and residual fibrosis were associated with vitality (*β* =  − 0.34, *p* = 0.008 and *β* =  − 0.41, *p* = 0.006, respectively) and general health improvement on SF-36 (*β* =  − 0.26, *p* = 0.0034; *β* =  − 0.28, *p* = 0.04, respectively).To further understand our findings, we compared the AFSS change in patients who had an increase in LAVI (adverse remodeling) versus a reduction in LAVI (positive remodeling). Although both groups experienced improvements in AFSS score, those with positive remodeling experienced a greater improvement (4.95 vs. 7.02, *p* = 0.006, Fig. 4).

## Discussion

This study demonstrates multiple novel findings regarding the association between atrial myopathy markers such as atrial fibrosis and atrial volume index with QoL improvements in persistent AF patients undergoing CA. Firstly, an inverse correlation between baseline and post-ablation LAVI and multiple QoL metrics was demonstrated. Higher atrial fibrosis at baseline and residual fibrosis post-ablation were associated with a lesser improvement in energy and fatigue levels in addition to a lower improvement in the patient’s perception of their general health. Moreover, there was a strong correlation between LAVI reduction post-ablation and QoL assessment on SF-36 and AFSS questionnaires in individuals with persistent AF, independent of age, comorbidities, and recurrence status.

Symptom relief represents the primary indication for AF ablation. However, post-ablation QoL assessment has proven controversial [20]. The degree to which AF symptoms impact patients varies significantly [21]. Prior studies have indicated that age, gender, comorbidities, and medications used are important factors contributing to AF symptoms [22–24]. In this study, we found that both atrial size and fibrosis assessed using LGE-MRI are associated with QoL outcomes. We found that post-ablation LAVI is associated with improvement in a multitude of QoL metrics including physical functioning, vitality, general health, health change, AF burden, shortness of breath at rest, and dizziness. The association between positive LA remodeling and QoL improvement highlights the importance of follow-up imaging in addition to functional and rhythm assessments in patients who have undergone AF ablation.

Prior studies have investigated left atrial remodeling following AF ablation. Jahnke et al. demonstrated progressive LA volume reduction following standard PVI with decreased recurrence [25]. Although Hof et al. found that conventional PVI is associated with LA volume reductions, there was no association between LAVI reduction and arrhythmia recurrence [26]. Beukema et al. assessed LA size at baseline and during follow-up using electroanatomic mapping and found a statistically significant relationship between medium-term procedural success and LA size [27]. Other studies have demonstrated that fibrosis-guided ablation in addition to conventional PVI is associated with more significant reductions in LA volume despite similar recurrence rates [10]. In the present study, we found that positive LA remodeling following AF ablation is significantly associated with improvements in QoL metrics, independent of arrhythmia recurrence, age, and gender. Moreover, fibrosis-guided ablation did lead to significantly more LAVI decrease but it did not translate to a significant difference in QoL or symptom improvement. Our findings are consistent with prior studies investigating the association between arrhythmia recurrence and QoL. For example, a STAR AF II substudy demonstrated post-ablation QoL improvements regardless of recurrence status [9]. Similarly, the CAPTAF study demonstrated significant improvements in QoL following ablation despite more than half of the study participants experiencing brief recurrences [28].

Atrial fibrosis is considered a hallmark of arrhythmogenic atrial tissue [29]. Atrial fibrosis leads to a structural and electrical heterogeneity important for the development and maintenance of atrial fibrillation through conduction slowing, heterogeneity, block, and reentry. These extra-pulmonary areas of fibrosis are hypothesized to lead to the decreased success rate associated with PVI, especially in persistent AF patients. Our results indicate that fibrosis leads also to attenuated improvement in both general health from the patient’s perspective and vitality levels. Patient symptoms can be considerably different between patients with paroxysmal or persistent AF. For example, while paroxysmal AF patients report palpitations more frequently, persistent AF patients usually report more fatigue-related symptoms. Hence, the association between fibrosis and improvement in vitality levels is not surprising and hints towards the possible role of advanced myopathy in the symptomatology of these patients. Moreover, residual fibrosis, i.e., fibrosis not covered by ablation-induced scar, was found to be also associated with these factors, providing complementary evidence for the possible role of fibrosis in these patients. Moreover, this may represent a signal that, in persistent AF patients, extra-pulmonary fibrosis coverage can lead to improved CA success. In DECAAF II, although there was an MRI fibrosis-guided arm, only 25% of fibrosis was covered, which serves as a limitation that needs to be explored.

Although our results are specific to patients with persistent AF, they do not contradict the established benefits of catheter ablation on QoL in patients with paroxysmal AF. Patients with persistent AF have lower success rates in achieving rhythm control with catheter ablation due to advanced myopathy. Our study demonstrates that despite suboptimal rhythm control compared to paroxysmal AF patients, catheter ablation still leads to significant QoL improvements in patients with persistent AF, comparable to those observed in paroxysmal AF. For example, a multicenter, single-arm study assessing the efficacy of multi-electrode balloon radiofrequency catheter ablation in 85 patients with paroxysmal AF showed significant improvement across all Atrial Fibrillation Effect on Quality-of-Life Questionnaire (AFEQT) subscale scores at both 6 months and 1 year post-ablation [30]. These findings align with results from a multicenter randomized controlled trial that compared second-line catheter ablation to antiarrhythmic drug therapy [31]. In this trial, patients who underwent catheter ablation demonstrated superior QoL improvement, as assessed by the SF-36 questionnaire, while antiarrhythmic drug therapy failed to achieve similar benefits. Notably, catheter ablation led to patients returning to QoL levels comparable to those of the healthy population. Thus, while rhythm control with catheter ablation is more effective in paroxysmal AF than in persistent AF, both patient populations experience significant benefits in terms of symptom relief and QoL improvement.

Our study has yielded insightful results, but it is important to acknowledge certain limitations. The retrospective design inherently limits our ability to establish causation. Variability across the participating centers may have influenced the study outcomes. Despite our efforts to standardize LGE-MRI protocols, the use of different MRI machines across sites could be a source of variability in our measurements. In terms of left atrial (LA) volume assessments, the absence of cine images in the DECAAF II imaging protocol meant that all measurements were conducted prior to atrial contraction. The lack of available data on the patients’ rhythm status at the time of MRI is another factor that could have influenced the accuracy of LA volume measurements. The QoL assessments employed, namely the SF-36 and AFSS, bring their own challenges. The SF-36, while extensively validated and commonly used in cardiology, is not tailored to atrial fibrillation, and may omit aspects unique to the condition. The AFSS, on the other hand, emphasizes symptom severity at the potential expense of the wider psychosocial impact of atrial fibrillation. Moreover, what constitutes a clinically meaningful change is difficult to determine given the absence of a gold standard to compare to. It is essential, therefore, to interpret our findings with a careful consideration of these methodological constraints.

## Conclusion

In conclusion, our study provides evidence that LA volume index, baseline fibrosis, residual fibrosis, and ablation-related structural remodeling are significantly associated with improvements in QoL for patients with persistent atrial fibrillation. LAVI decrease is associated with symptomatic improvement regardless of recurrence.

## Supplementary Information

Below is the link to the electronic supplementary material.

Supplementary file1 (DOCX 17 KB)

## Abbreviations

AF: Atrial fibrillation
CA: Catheter ablation
QoL: Quality of life
LGE-MRI: Late-gadolinium enhancement cardiac magnetic resonance imaging
PVI: Pulmonary vein isolation
ECG: Electrocardiography
LA: Left atrium
