# Supplementary material for: Impact of left atrial myopathy and post-ablation remodeling on quality of life: a DECAAF II sub-analysis
Source: J Interv Card Electrophysiol. 2025 Feb 3;68(6):1225–34. doi: 10.1007/s10840-025-02002-1 (PMC12399685; doi:10.1007/s10840-025-02002-1)
Supplement: Supplementary file 1 — Supplementary file1 (DOCX 17 KB) [file 10840_2025_2002_MOESM1_ESM.docx]

Supplemental Material

Table 1: Univariable Analysis of CMR indices and QoL Outcomes on the AFSS

| Variable/Outcome | LAVI Change | Baseline Fibrosis | Residual Fibrosis |
| --- | --- | --- | --- |
| Global Well Being Improvement in AFSS | β=-0.016  p-value=0.018 | β= 0.015  p-value= 0.191 | β=0.01  p-value= 0.310 |
| AF Frequency Improvement in AFSS | β=0.108  p-value<0.001 | β=0.02  p- value = 0.376 | β=0.01  p-value= 0.791 |
| AF Duration Improvement in AFSS | β=0.001  p-value=0.962 | β=-0.05  p-value=0.142 | β=-0.07  p-value=0.069 |
| AF Severity Improvement in AFSS | β=-0.018  p-value=0.064 | β=-0.01  p-value= 0.487 | β=-0.01  p-value=0.541 |
| AF Burden Improvement in AFSS | β=0.152  p-value<0.001 | β=0.008  p-value=0.856 | β=-0.02  p-value=0.735 |
| Palpitations Improvement in AFSS | β=0.011  p-value=0.068 | β=-0.014  p-value=0.178 | β=-0.02  p-value=0.192 |
| Improvement in Shortness of Breath at rest | β=0.011  p-value=0.039 | β=0.001  p-value= 0.853 | β=0.003  p-value=0.733 |
| Improvement in Shortness of Breath at activity | β=0.021  p-value<0.001 | β=0.001  p-value= 0.922 | Β=0.0002  p-value= 0.979 |
| Improvement in Exercise Tolerance | β=0.022  p-value<0.001 | β=-0.004  p-value=0.668 | β=-0.006  p-value=0.553 |
| Improvement in Fatigue at rest | β=0.009  p-value= 0.084 | β=-0.009  p-value= 0.261 | β=-0.0014  p-value=0.142 |
| Improvement in Dizziness at rest | β=0.004  p-value=0.430 | β=-0.01  p-value=0.163 | β=-0.011  p-value= 0.243 |
| Improvement in Chest Pain at rest | β=0.001  p-value=0.832 | β=0.002  p-value= 0.725 | β=0.006  p-value= 0.502 |
| Total AFSS Improvement | β=0.086  p-value= 0.001 | β=-0.03  p-value=0.390 | β=-0.0447  p-value=0.386 |

*Legend: AF: Atrial Fibrillation; AFSS: Atrial Fibrillation Severity Scale*

Table 2: Univariable Analysis and QoL outcomes on the SF-36

| Variable/Outcome | LAVI Change | Baseline Fibrosis | Residual Fibrosis |
| --- | --- | --- | --- |
| Physical Functioning Improvement on SF-36 | β=0.27  p-value=0.001 | β=-0.159  p-value=0.266 | β=-.02  p-value= 0.204 |
| Improvement in Limitations due to physical Health on SF-36 | β=0.53  p-value<0.001 | β=-0.069  p-value=0.790 | β=-0.10  p-value=0.73 |
| Improvement in Limitations due to Emotional Problems on SF-36 | β=0.131  p-value= 0.318 | β=0.028  p-value= 0.898 | β=0.06  p-value= 0.814 |
| Improvement in Energy and Fatigue on SF-36 | β=0.197  p-value= 0.01 | β=-0.34  p-value= 0.008 | β=-0.41  p-value= 0.006 |
| Improvement in Emotional Well-being on SF-36 | β=0.100  p-value= 0.083 | β=-0.01  p-value= 0.331 | β=-0.13  p-value= 0.251 |
| Improvement in Social Functioning on SF-36 | β=0.14  p-value= 0.076 | β=-0.13  p-value= 0.338 | β=-0.22  p-value= 0.149 |
| Improvement in Pain on SF-36 | β=0.111  p-value= 0.199 | β=0.13  p-value= 0.374 | β=0.14  p-value= 0.373 |
| Improvement in General Health | β=0.134  p-value=0.062 | β=-0.26  p-value=0.034 | β=-0.28  p-value=0.04 |
| Improvement in Health Change | β=0.519  p-value<0.001 | β=0.27  p-value=0.151 | β=0.3  p-value=0.163 |

*Legend: LAVI: Left atrial Volume Index; SF-36: 36-Item Short Form Survey*
